# Prediction of suicidal ideation and attempt in 9 and 10 year-old children using transdiagnostic risk features

**DOI:** 10.1371/journal.pone.0252114

**Published:** 2021-05-25

**Authors:** Gareth Harman, Dakota Kliamovich, Angelica M. Morales, Sydney Gilbert, Deanna M. Barch, Michael A. Mooney, Sarah W. Feldstein Ewing, Damien A. Fair, Bonnie J. Nagel

**Affiliations:** 1 Department of Medical Informatics & Computational Epidemiology, Oregon Health & Science University, Portland, OR, United States of America; 2 Department of Psychiatry, Oregon Health & Science University, Portland, OR, United States of America; 3 Department of Behavioral Neuroscience, Oregon Health & Science University, Portland, OR, United States of America; 4 Departments of Psychological & Brain Sciences, Psychiatry, Radiology, Washington University, St. Louis, MO, United States of America; 5 Department of Psychology, University of Rhode Island, Kingston, RI, United States of America; 6 Department of Pediatrics and Institute of Child Development, University of Minnesota, Minneapolis, MN, United States of America; Case Western Reserve University, UNITED STATES

## Abstract

The objective of the current study was to build predictive models for suicidal ideation in a sample of children aged 9–10 using features previously implicated in risk among older adolescent and adult populations. This case-control analysis utilized baseline data from the Adolescent Brain and Cognitive Development (ABCD) Study, collected from 21 research sites across the United States (N = 11,369). Several regression and ensemble learning models were compared on their ability to classify individuals with suicidal ideation and/or attempt from healthy controls, as assessed by the Kiddie Schedule for Affective Disorders and Schizophrenia–Present and Lifetime Version. When comparing control participants (mean age: 9.92±0.62 years; 4944 girls [49%]) to participants with suicidal ideation (mean age: 9.89±0.63 years; 451 girls [40%]), both logistic regression with feature selection and elastic net without feature selection predicted suicidal ideation with an AUC of 0.70 (CI 95%: 0.70–0.71). The random forest with feature selection trained to predict suicidal ideation predicted a holdout set of children with a history of suicidal ideation and attempt (mean age: 9.96±0.62 years; 79 girls [41%]) from controls with an AUC of 0.77 (CI 95%: 0.76–0.77). Important features from these models included feelings of loneliness and worthlessness, impulsivity, prodromal psychosis symptoms, and behavioral problems. This investigation provided an unprecedented opportunity to identify suicide risk in youth. The use of machine learning to examine a large number of predictors spanning a variety of domains provides novel insight into transdiagnostic factors important for risk classification.

## Introduction

Prevalence of youth suicide is a serious public health concern, prompting substantial research attention in recent years. As of 2017, suicide was the second leading cause of death for individuals between the ages of 10 and 24 [1], and although the number of fatalities due to suicide in this youngest age bracket falls below that of several older groups, the prevalence of suicide deaths for 10–14 year-olds has nearly tripled since 2007 [2]. Such an alarming surge in preventable deaths, particularly among youth, highlights the gravity of this issue, as well as the urgent need for better, more innovative risk screening and early intervention strategies.

Many of the known risk factors for suicidal thoughts and behaviors (STBs) have been gleaned from studies of older adolescent (13–18 years) or adult populations due to the rapid increase in prevalence in the late teens and early twenties [3]. A comprehensive meta-analysis of the existing literature demonstrated that current psychopathology (specifically depression), prior history of suicidal ideation (SI) and/or attempt (SA), psychiatric hospitalization, and stressful or traumatic life events were consistently identified as predisposing factors [4]. Although evidence suggests that STBs begin to emerge much earlier in development [5], it remains unclear to what extent risk factors identified in adolescents and adults generalize to preadolescent children [6]. For example, some foundational studies examining younger populations (7–12 years) identified psychopathology as a risk factor for STBs [7], but this is complicated by the fact that diagnostic indicators of risk are often not yet fully evident in childhood [8]. Diagnostic categories also include a variety of symptoms that vary along continuums; thus, heterogeneity may be obscured by focusing on the presence or absence of a diagnosis. Many symptoms span across diagnostic categories; therefore, targeting prevention and intervention efforts at specific symptoms rather than disorders may prove more effective.

Another obstacle to reliable suicide risk prediction may be a reliance on traditional statistical approaches to model simple relationships between a circumscribed set of risk factors. While recent studies in children (9–10 years) employing this approach have identified family-related factors [9, 10], and peer victimization/bullying [11] as increasing risk for STBs, the use of machine learning (ML) may substantially improve prediction accuracy. ML algorithms are optimally suited to investigate high-dimensional data and handle nuanced interactions between predictive features. In adults, ML approaches have improved estimation accuracy for future STBs dramatically (AUC 0.71–0.89) [12] when compared to conventional methods, which perform at or near chance levels (AUC 0.57–0.58) [4]. However, these more complex models are often difficult to interpret, and therefore potentially less amenable to adaptation into clinical screening tools.

In the present study, we built and compared several models, ranging in complexity from logistic regression to the random forest, to identify consistent predictors of STBs which could be used to inform risk screening. We aimed to examine a range of transdiagnostic risk factors for SI and SA by capitalizing on the wealth of data from the Adolescent Brain and Cognitive Development (ABCD) Study [13], including a comprehensive battery of item-level features and summary scores. Although several studies have explored risk factors related to suicidality in this sample, they have either focused on a selective set of predictors (e.g., family conflict, parental monitoring, psychopathology, neurocognition) or utilized simple modeling strategies (e.g., logistic regression, mixed effects models) [9, 10, 14]. Here, by considering several predictive algorithms and a wide array of features simultaneously, we sought to strike a balance between predictive power and model interpretability. Due to the comparatively low incidence but critical repercussions of SA, we also tested the ability of a predictive model trained using SI alone to classify co-occurring SI and SA, which has yet to be done in the ABCD sample.

## Methods

### Participants

This study included 11,369 of the 11,874 participant families (child and parent/caregiver dyads) enrolled in the 21-site ABCD Study (https://abcdstudy.org, Release 2.0). All caregivers and children provided written informed consent/assent for participation. All study procedures were approved by an Institutional Review Board. As of 2021, 20 of the 21 ABCD research sites rely on a centralized single Institutional Review Board at the University of California, San Diego, with the exception of the site at Washington University in St. Louis, which utilizes their local Institutional Review Board for approval. Sampling, recruitment, inclusionary/exclusionary criteria, and assessment measures for the ABCD Study have been described in detail previously [13, 15, 16]. Participants were excluded from the present analysis if they were missing ≥15% of features used as input (S1 Table in S1 File). Based on either caregiver or child endorsements on the Kiddie Schedule for Affective Disorders and Schizophrenia–Present and Lifetime Version using *Diagnostic and Statistical Manual of Mental Disorders (Fifth Edition)* criteria (K-SADS-PL, DSM-5, computerized) [17], participants were classified as controls (n = 10,060), having active suicidal ideation but no history of or current attempts (SI; n = 1,116), or having attempted suicide (SA/SI; n = 193). Neither endorsements of passive suicidal ideation (i.e., current or previous wish to be dead, or belief that they would be better off dead) nor self-injurious behavior in the absence of suicidal intent (i.e., current or previous engagement in purposeful behaviors to experience physical injury for reasons other than dying by suicide) were included in this study. Although sometimes related to risk for suicidality [18], non-suicidal self-injury (NSSI) is thought to be a distinct condition with different etiology from suicidality [19]. Likewise, passive suicidal ideation may be distinct from active suicidality, and could reflect development surrounding the concept of death in this age group [20].

### Predictive variable selection

In total, there were 323 features used as predictors to classify individuals with suicidal ideation from controls. These features were selected to include previously implicated risk factors associated with child and adolescent SI/SA (e.g., [9, 10, 21–25]) with an eye toward capitalizing on extant ABCD data collection. This set of inputs included a mix of both item-level and summary score data from each of the following assessment domains: demographics [26], neurocognitive assessments [27], mental and physical health measures [26, 28], and culture/environmental variables [29] (S1 Table in S1 File). To maximize the accessibility of transdiagnostic risk assessment across a variety of settings, diagnostic items from the K-SADS-PL, DSM-5 were generally not included as predictors. Only two features were retained from this measure, as they were uncollected elsewhere, which included questions regarding sexual orientation and experiences of trauma. Features missing ≥15% of observations were removed from the dataset, as were participants missing ≥15% of the input features. The remaining missingness was imputed using random forests from the R package *randomForestSRC* [30].

### Feature coding

Several features underwent transformations of either aggregation or numeric recoding in order to be compatible with the methods utilized in this study. First, medications were coded into binary categories of “yes” or “no” for taking any depression-, ADHD-, or opioid-related medications. These categories were determined by custom scripts querying the RxNorm database via its RESTful API. Further details can be found on Gitlab (https://gitlab.com/gareth_harman/abcd_meds). Secondly, due to the substantial number of features related to hobbies/interests (>20), summary features were generated to describe the raw counts of activities endorsed in each of three categories (“physical”, “creative”, and “other”). Physical activities included all sports, creative activities included music and art, and other activities included hobbies, such as collecting. Finally, because all of the algorithms used here, save for the random forest, require only numeric input features, all categorical features were recast into numeric form. Features with inherent ordinal structure were recast into integers incrementing by 1 for each level. While it is not possible to assume equal distance between levels of ordinal features, this circumvents issues surrounding feature inflation (i.e., creating many one-hot encoded features for variables with multiple levels) and retains the ordinal nature of the feature. Features such as race/ethnicity, gender, highest education achieved by either parent, household income, parents’ marital status, and ABCD data collection site were all one-hot encoded.

### Data partitioning

**Fig 1:**
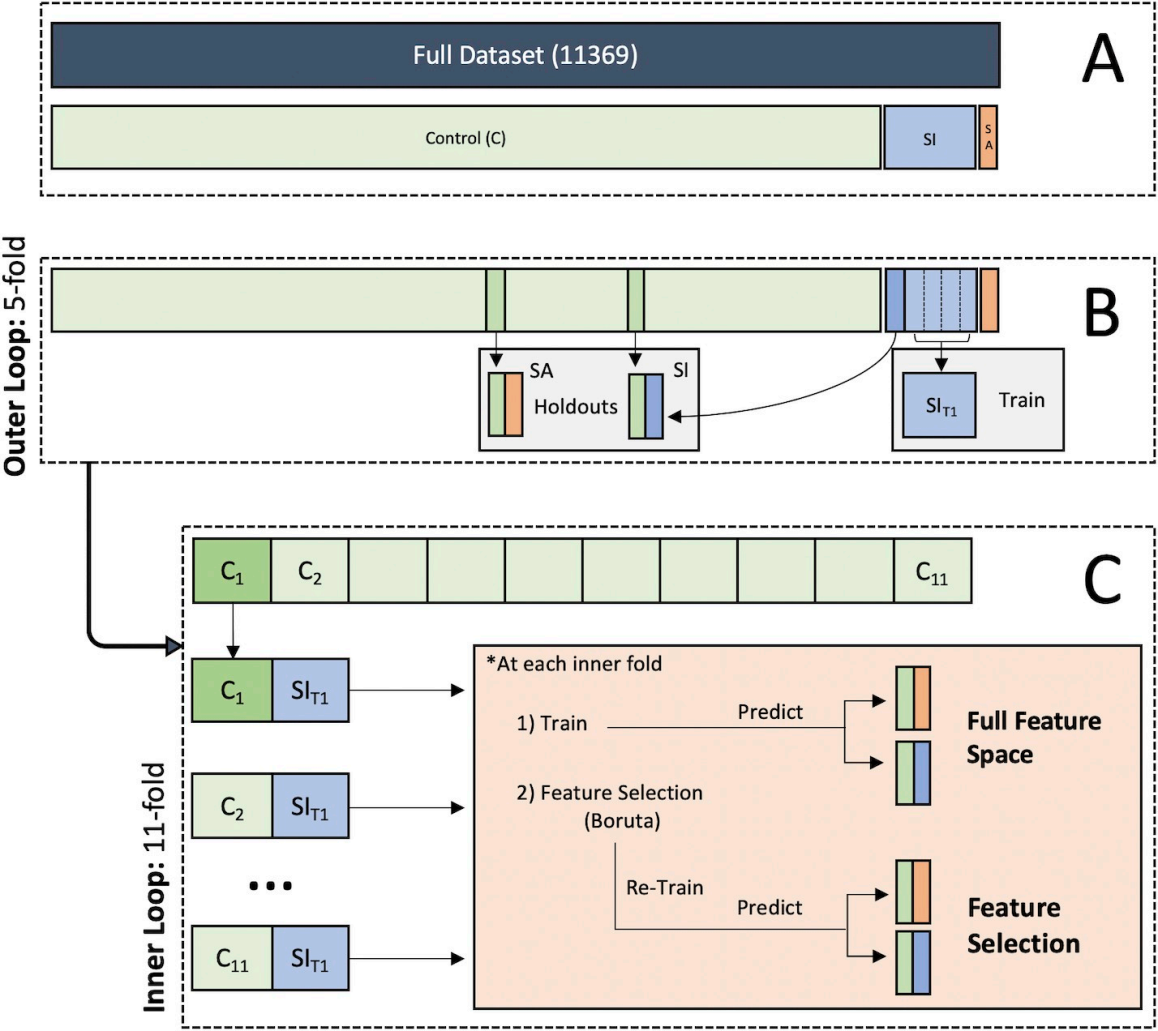
Nested cross-fold validation data partitioning scheme. **A.** First, data were partitioned into respective class labels. **B.** Next, the outer loop split the suicidal ideation (SI) class into five folds, setting aside one fold and a randomly subsampled set of controls as a holdout test set for each iteration. The group of individuals endorsing concomitant suicidal ideation and attempt (SA), along with another randomly subsampled set of controls were also set aside as a holdout test set. **C.** The inner loop then divided the remaining control sample into 11 folds (N_Control_/N_SI_) to combine with the SI training set to create class balanced datasets for training. These balanced training sets circumvent common class imbalance issues in machine learning, while also mitigating sampling bias induced by techniques such as down-sampling. Therefore, the reported performance for each model spanned the 55 total folds for each set of inner (11-folds) and outer folds (5-folds).

For model training and evaluation, we employed a nested cross-fold validation partitioning scheme (Fig 1). Initially, the data were grouped by respective class labels (control, SI alone, concomitant SI and SA; Fig 1A). The *outer loop* split the SI class into five folds, setting aside one fold and a randomly subsampled set of controls as a holdout test set for each iteration. The entire sample of individuals endorsing co-occurring SI and SA, along with another randomly subsampled set of controls, was set aside as an additional holdout test set for each iteration (Fig 1B). The remaining controls and SI participants were retained in the *inner loop* for use in model training. Within the inner loop, the majority class (controls) was split into 11 folds to create folds that were approximately the same size as the SI class being used for training (Fig 1C). Crucially, this strategy allowed for the construction of training sets that had balanced class sizes, thus mitigating the issue of algorithms favoring the majority class when addressing classification problems with highly imbalanced datasets [31]. In addition to dealing with class imbalance, the repetition of training provides a distribution of performance given unique subsets of controls and participants endorsing SI. This method reduces the likelihood of sampling bias compared to more traditional down-sampling approaches which train a model on a single, randomly-selected (but potentially less representative) subset of the data [32].

### Feature selection

In addition to building models using the entire set of 323 features, we generated a more constrained feature space for training using the Boruta feature selection algorithm as implemented in the *Boruta* R package [33]. In short, the Boruta algorithm creates a “shadow” variable for each existing input variable in the dataset and permutes the labels. Random forests (RF) are then trained using all features (both original and shadow) and permutation importance scores are computed for each feature. The Boruta algorithm identifies the shadow feature with the highest permutation importance and retains all original features that have permutation importance scores that are *significantly greater* than this most important shadow feature. This approach provides an empirical process for determining input features that are significantly more informative than a best-performing known *noise* parameter for a given classification task.

### Model training

The RF, a supervised ensemble learning method commonly used for both classification and regression problems, was used to predict suicidal ideation via the *randomForest* package in R [34]. For a classification task, the RF builds a specified number of decision trees where each split (decision) is based on the feature, out of a randomly selected subset of features, that best partitions the data according to class label. To justify the added complexity of this model, as well as the limitations of interpretability, we compared the performance of this method to both logistic and penalized regression methods, including ridge, lasso, and elastic net using the R package *glmnet* [35]. All models were trained at each of the five outer and 11 inner folds (55 total folds), then used to predict the unique holdout sets of both SI and co-occurring SI/SA. Subsequently, the Boruta feature selection method was employed to identify the most informative features to be retained out of the original 323 inputs. The models were then re-trained in this constrained feature space and used to predict SI and SI/SA holdout sets.

### Sibling effects

To maximize the size of each test set, we elected to include siblings in these analyses. However, it is important to note that when conducting analyses with biological data (e.g., genomic or neuroimaging data), such results may be biased by including observations which are not truly independent. In the case of the predictive methods included here, the concern with including sibling pairs is an overestimation of predictive accuracy. For example, if one sibling is included in the training set and one in the test set, it could be argued that the familial similarity of these observations falsely inflates test performance. Thus, to assess how the inclusion of siblings in the test sets impacted predictive ability, we conducted a post-hoc analyses in which we removed subjects from each test set if they had a family member in the given training set, and then re-evaluated performance. Because simply altering the size of a test set could affect performance, we also generated test sets in which an equal number of participants were removed at random to appropriately compare predictive accuracy to the test sets with siblings removed. Performance was evaluated on each test set (siblings removed vs. subjects removed at random), and ANOVAs were used to examine any significant differences between the test sets.

## Results

**Fig 2:**
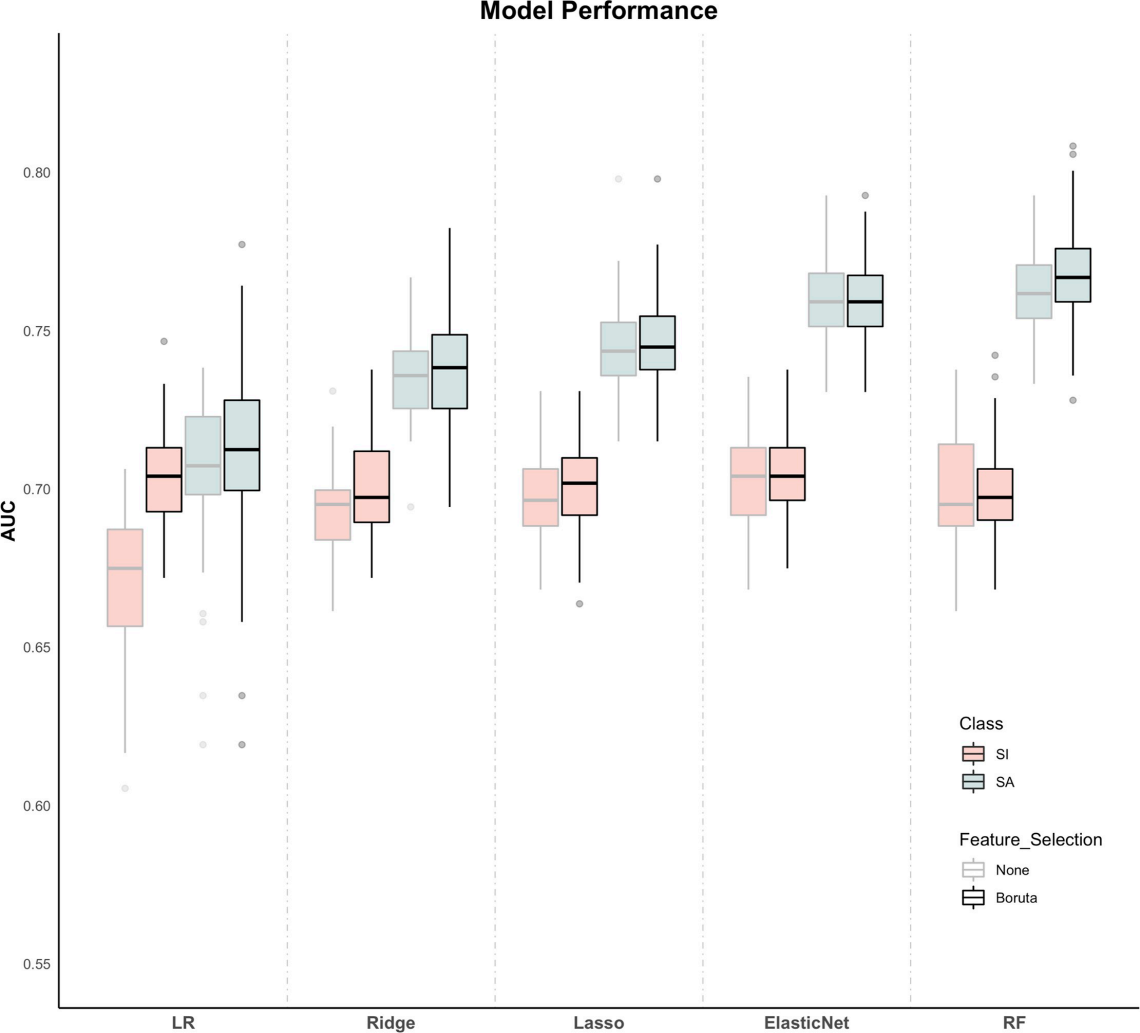
Model performance. Overall model performance in predicting both suicidal ideation (SI) and concomitant suicidal ideation and attempt (SA) using each method with and without Boruta feature selection. Logistic regression with feature selection and elastic-net without feature selection were the top performing models for classifying SI from controls (AUC = 0.70; CI 95%: 0.70–0.71), but did not perform significantly better than any other model trained after feature selection. The random forest with feature selection, trained only on SI vs. controls, was able to distinguish a smaller holdout test set of individuals endorsing both SI and SA from controls (AUC = 0.77; CI 95%: 0.76–0.77).

**Fig 3:**
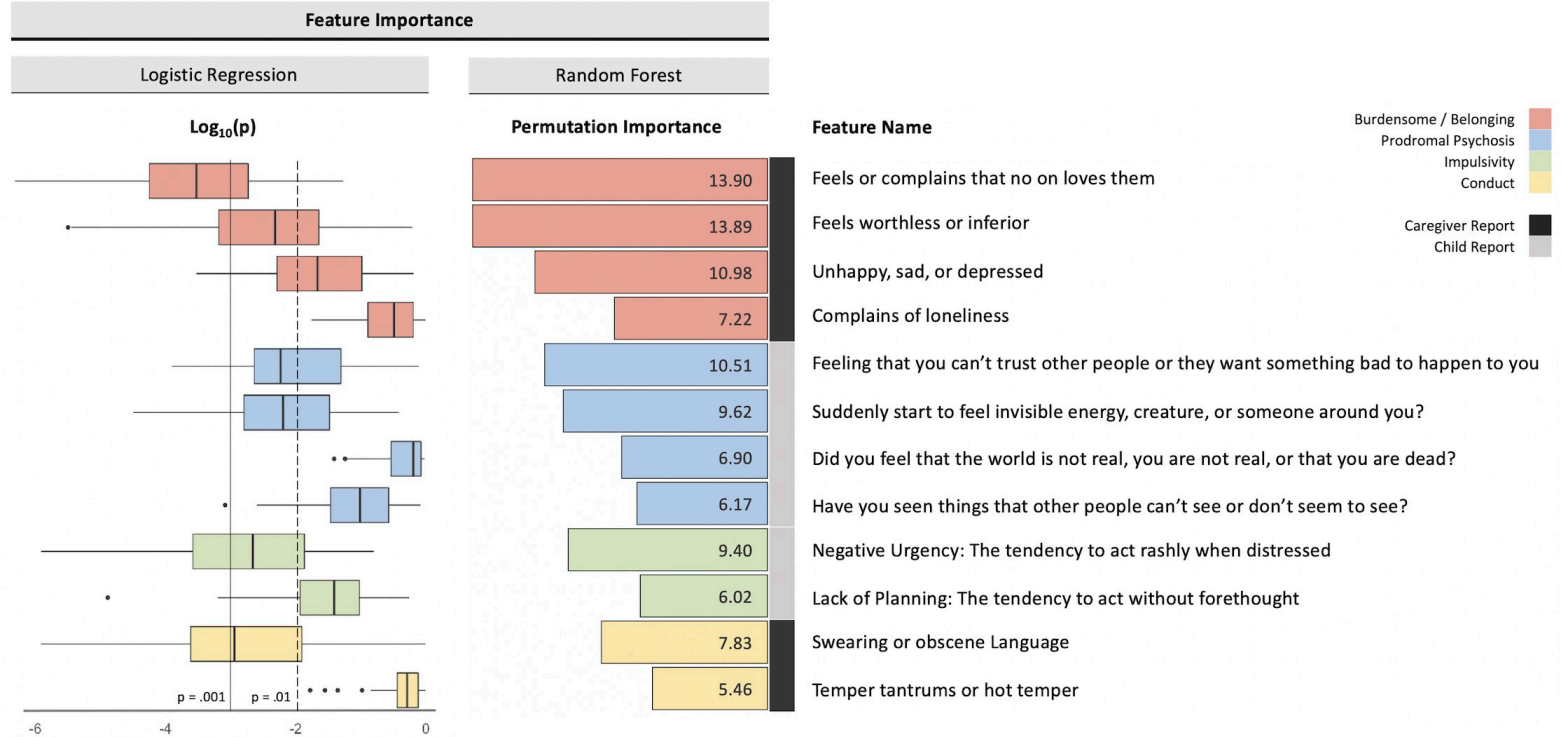
Feature importance. Boxplots display the log_10_ p-value of the most important features from each fold of training for logistic regression after Boruta feature selection. Vertical lines represent different levels of significance (p < 0.01, p < 0.001). Bar plots show the mean permutation importance of features identified as being more important than a known noise feature at every fold of training for the random forest. In both cases, feeling unloved, loneliness, measures of impulsivity, prodromal symptoms, and behavioral problems were important. Associated colors represent the theoretical construct for each feature, and whether that feature was derived from a caregiver- or child-report measure.

Participant demographics are presented in Table 1. Among the models trained using all features for the classification of SI and control groups, the elastic net performed the best with an AUC of 0.70 (CI 95%: 0.70–0.71, Fig 2). Model training after feature selection significantly improved the performance of logistic regression and ridge regression in classifying SI from controls (p < 0.001), but did not improve the performance of lasso regression (p = 0.32), elastic net regression (p = 0.57), or random forest (p = 0.67). The best model for predicting SI after feature selection was logistic regression, which also had an AUC of 0.70 (CI 95%: 0.70–0.71, Fig 2). The features that were identified as important at every fold of training, determined from the Boruta feature selection algorithm, included components of loneliness, impulsivity, feelings of being unloved, prodromal psychosis symptoms, and conduct problems (Fig 3). A nearly identical set of features was found to be significant at every fold during training for the logistic regression model after feature selection (Fig 3). Additionally, these important features did not differ between individuals with internalizing vs. externalizing disorders, underscoring their transdiagnostic nature (all p’s > 0.01; S1 Fig and S2 Table in S1 File).

**Table 1:** Participant demographics.

	CTRL		SI		SA		CTRL/SI	CTRL/SA	SI/SA
N	10060		1116		193				
Age	9.92 ± .62		9.89 ± .63		9.96 ± .62				
Age (SD)	0.62		0.63		0.62				
Male	5116	0.51	665	0.60	114	0.59	***		
Female	4944	0.49	451	0.40	79	0.41			
	N	%	N	%	N	%			
	**Race / Ethnicity**								
Asian	214	0.02	24	0.02	3	0.02			
Black	1534	0.15	149	0.13	46	0.24			*
Hispanic	2048	0.20	215	0.19	47	0.24			
White	5250	0.52	589	0.53	74	0.38		**	*
Other	968	0.10	138	0.12	23	0.12			
Parents Married	6873	0.68	703	0.63	97	0.50	*	***	**
	**Parent Highest Education**								
< Highschool	508	0.05	49	0.04	10	0.05			
Highschool / GED	965	0.10	94	0.08	27	0.14			
Some College	2542	0.25	316	0.28	72	0.37		**	
Bachelors	2562	0.25	280	0.25	48	0.25			
Graduate	3474	0.35	375	0.34	36	0.19		***	**
	**Total Household Income**								
Income < = 50k	2677	0.27	304	0.27	89	0.46		***	***
50k < Income < 100k	2582	0.26	311	0.28	48	0.25			
Income > = 100k	3945	0.39	396	0.35	41	0.21		***	**

Several demographic elements differed significantly between control, suicidal ideation (SI), and co-occurring suicidal ideation and attempt (SA) groups. Sex, race/ethnicity, parental marital status, highest level of parental education, and total household income, were all significantly different between at least two of the groups. Level of significance after Bonferroni correction denoted by ***, **, and * (p < .001, p < .01, and p < .05).

When predicting co-occurring SI and SA using the models trained to distinguish between SI alone and controls, the random forest using all features performed the best with an AUC of 0.77 (CI 95%: 0.76–0.77). Feature selection did not significantly improve the performance of any models on the co-occurring SI and SA holdout set (all p’s > 0.1). Furthermore, this random forest, trained using only SI, performed significantly better than the best performing model (random forest with feature selection) trained using this smaller group of individuals with concomitant SI and SA from controls, which had AUC of 0.72 (CI 95%: 0.71–0.73). There were no differences between the test sets with sibling pairs removed and subjects removed at random, thus providing justification for the initial decision to include siblings (S2 Fig in S1 File).

## Discussion

Capitalizing on the rich data collection and large sample size of the ABCD Study, this study trained a series of predictive models, using previously implicated risk features for STBs among adolescent and adult populations, to examine whether it is possible to distinguish children endorsing SI or SI/SA from controls. Overall, we found that logistic regression with feature selection and elastic net regression without feature selection emerged as top-performing models for classifying SI participants from controls. Moreover, we were able to use the logic derived from models trained to classify children with SI alone to a holdout test set of children with concomitant SI and SA with substantially improved performance. These results reaffirm collective concern about the emergence of STBs in young children, and also demonstrate the ability to distinguish vulnerable children using a set of items from questionnaires that can be administered easily without the need for a full diagnostic interview.

The important features identified by the Boruta feature selection algorithm were also determined to be significant by logistic regression and align with prior literature [4, 36]. Several prominent ideation-to-action theories of suicide, such as the interpersonal theory of suicide, posit that a combination of *thwarted belongingness* (i.e., feelings of being alone without any support) and *perceived burdensomeness* (i.e., feelings of being a burden to others) are core motivators for suicidal ideation [37, 38]. In the present study, we found that feelings of loneliness, or of worthlessness and inferiority, were among the most important features for classifying children with SI from controls, which aligns well with the concepts of thwarted belongingness and perceived burdensomeness. In addition, we found negative urgency and impulsivity to also be important features, which may suggest that a propensity to act without forethought helps delineate pre-adolescent children with STBs from controls. Though impulsivity does not itself indicate an *acquired capability* for suicide (the transition from ideation to potentially lethal action), it is a known predictor of engaging in self-injurious and suicidal behavior [37–40].

By employing a series of ML approaches that are optimally suited to handle high-dimensional data, we have been able to investigate a substantially larger number of potentially predictive features than previous studies examining STBs in the ABCD sample [9, 10]. For example, while both internalizing and externalizing composite scores from the Child Behavior Checklist [41] were found to be significantly associated with suicidality in prior work [9, 10], our approach allows for item-level decomposition of this measure. Specifically, we demonstrated that transdiagnostic CBCL items pertaining to depressed mood (Q12: Complains of loneliness, Q33: Feels or complains that no one loves them, Q35: Feels worthless or inferior, Q103: Unhappy, sad, or depressed) and rule-breaking/aggressive behavior (Q90: Swearing or obscene language, Q95: Temper tantrums or hot temper) were among the most informative features for distinguishing children with SI from controls. Interestingly, these results seem to parallel the two pathways to suicidality most heavily researched within adolescent populations [42]. In one pathway, it is suggested that suicidality is motivated by depression and a desire to die, while in the other, suicidality is integrally tied to frustration, reactive aggression, and poor impulse control, particularly in response to stressful life events [7, 42–44]. Furthermore, among the feature set we utilized were variables related to prodromal psychosis symptoms, which have been previously unstudied with respect to STBs in the ABCD sample. Importantly, there is a substantial body of literature demonstrating elevated levels of STBs among children and adolescents at high clinical risk for psychosis [45, 46], highlighting the importance of including such variables in risk analyses. While it is tempting to over-interpret individual predictor importance, we advocate that these findings be considered in the context of the respective algorithm. For example, Boruta constructs random forests comprised of ensembles of decision tress with nodes representing splitting rules for features in each tree. Therefore, the calculated permutation importance values are themselves a function of all previous feature splits in each decision tree. This may explain why certain features, such as those relating to parental monitoring and family environment, found to be significantly associated with suicidality in other studies utilizing ABCD data [9, 10], did not appear in the top predictive features of the present analysis. However, it is worth noting that the important features we have identified were recognized at *every one* of the 55 unique folds, and we found substantial overlap between the features identified by Boruta and by logistic regression after feature selection, which provides a measure of confidence. In sum, it is likely that STBs emerge from the contribution and interaction of these important elements, none of which precipitate suicidality in isolation, but together create a “perfect storm” of risk. From this perspective, the advantage of using ML to model these high-dimensional feature spaces and potential interactions between risk features is clear, even though we are unable to interpret elements of classical hypothesis testing, such as effect sizes. Furthermore, our work has demonstrated that combining regularized and ensemble methods with simpler models (e.g., logistic regression) and feature selection can lend greater insight and interpretability to aid in clinical translation.

Importantly, this study demonstrated that logic built to classify individuals with only SI from controls holds true for classifying a holdout set of individuals with both SI and SA with improved performance. Not only does this serve as an internal replication of our findings, but it also suggests that these features are particularly relevant for distinguishing individuals with concomitant SI and SA from controls, as evidenced by the improvement in AUC. Additionally, this approach holds promise for developing new methods of risk assessment. Future work should aim to clarify which features differentially predict SI and SA to understand the transition from ideation to action.

The present study has several limitations. First, we elected not to include participants who only endorsed non-suicidal self-injury (NSSI) or passive suicidal ideation (i.e., wishful thinking about death) in either group. Although there is evidence to suggest that NSSI is a distinct clinical syndrome with different etiology and predisposing risk factors relative to STBs [19], self-injurious behavior may be an important risk factor precipitating the acquired capability for suicide [18]. Thus, our narrowed definitions of SI and SA would not capture this. Second, psychiatric diagnoses were not included as input features for any of the models. While there is strong evidence associating current psychopathology with risk for STBs [7], structured diagnostic interviews can be costly and inaccessible. By focusing instead on granular, potentially transdiagnostic risk features, our findings may be more practical in terms of translational risk assessment and intervention. This approach also circumvents the use of arbitrary diagnostic thresholds. Third, we observed low correspondence between caregiver- and child-report with regard to STBs. Although this is not uncommon [47], we argue that endorsement of ideation or attempt from either caregiver or child should be considered seriously and included participants who met criteria for SI or SA on either caregiver- or child-report measures. However, future work should try to disentangle these potentially unique risk profiles, as it is possible that the features for distinguishing SI and SA could look different depending on the informant. Finally, it is not possible to determine causal relationships from this cross-sectional analysis. As the ABCD cohort continues to be monitored through adolescence and into young adulthood, researchers will have the opportunity to assess whether these models can prospectively predict the emergence of STBs. While ABCD already represents an unprecedentedly large sample from which to examine predictors of STBs in children, subsequent years of data will also increase the statistical power for such analyses detect meaningful effects [48].

## Conclusion

In conclusion, this study provides evidence for the ability to identify children between the ages of 9 and 10 endorsing SI from healthy controls, as well as those with concomitant SI and SA from healthy controls. Furthermore, many of the features identified as important from these models have been previously implicated in risk for SI and SA among older adolescent and adult populations, including feelings of loneliness and worthlessness, impulsivity, prodromal psychosis symptoms, and behavioral problems. Future analyses with longitudinal and multimodal data may provide additional predictive power.

## Supporting information

S1 File (DOCX)
