# Supplementary material for: Prediction of suicidal ideation and attempt in 9 and 10 year-old children using transdiagnostic risk features
Source: PLoS One. 2021 May 25;16(5):e0252114. doi: 10.1371/journal.pone.0252114 (PMC8148349; doi:10.1371/journal.pone.0252114)

**S1 File**

**Supplemental Table 1 (ST1).** *Input features used for model building.*

**Supplemental Table 2 (ST2).** *Features used to generate internalizing and externalizing disorder labels.*

**Supplemental Figure 1 (****SF1).** *Transdiagnostic validation of top predictive features.*

**Supplemental Figure 2 (SF2).** *Performance differences with sibling or random subject removal.*

**S1 Table.** *Input features for model building.*

| **Variable Name (ABCD)** | **Variable Description (ABCD)** | **Number of Missing Responses** | **Number Refused to Answer** |
| --- | --- | --- | --- |
| gender | Sex of the subject | 0 | 0 |
| interview_age | Age in months at the time of the interview/test/sampling/imaging. | 0 | 0 |
| crpbi_acceptance_caregiver12 | Second caregiver. Makes me feel better after talking over my worries with him/her. | 918 | 0 |
| crpbi_acceptance_caregiver13 | Second caregiver. Smiles at me very often. | 917 | 0 |
| crpbi_acceptance_caregiver14 | Second caregiver. Is able to make me feel better when I am upset. | 920 | 0 |
| crpbi_acceptance_caregiver15 | Second caregiver. Believes in showing his/her love for me. | 918 | 0 |
| crpbi_acceptance_caregiver16 | Second caregiver. Is easy to talk to. | 920 | 0 |
| devhx_12a_born_premature_p | Was the child born prematurely? | 146 | 0 |
| devhx_6_pregnancy_planned_p | Was your pregnancy with this child a planned pregnancy? | 267 | 0 |
| fam_history_5_yes_no | Has ANY blood relative of your child ever had any problems due to drugs, such as: Marital separation or divorce; Laid off or fired from work; Arrests or DUIs; Drugs harmed their health; In a drug treatment program; Suspended or expelled from school 2 or more times; Isolated self from family, caused arguments or were high a lot. | 319 | 7 |
| fam_history_6_yes_no | Has ANY blood relative of your child ever suffered from depression, that is, have they felt so low for a period of at least two weeks that they hardly ate or slept or couldn't work or do whatever they usually do? | 507 | 5 |
| famhx_4_p | Has ANY blood relative of your child ever had any problems due to alcohol, such as: Marital separation or divorce; Laid off or fired from work; Arrests or DUIs; Alcohol harmed their health; In an alcohol treatment program; Suspended or expelled from school 2 or more times; Isolated self from family, caused arguments or were drunk a lot. | 323 | 8 |
| famhx_11_yes_no_p | Has ANY blood relative of your child ever been to a doctor or a counselor about any emotional or mental problems, or problems with alcohol or drugs? | 450 | 12 |
| famhx_13_yes_no_p | Has ANY blood relative of your child ever attempted or committed suicide? | 511 | 9 |
| famhx_8_yes_no_p | Has ANY blood relative of your child ever had a period lasting six months when they saw visions or heard voices or thought people were spying on them or plotting against them? | 447 | 3 |
| lmt_scr_efficiency | Little Man Task Efficiency. | 348 | 0 |
| resiliency_5a | How many friends that are boys do you have? | 29 | 0 |
| resiliency_5b | How many CLOSE friends that are boys do you have? | 27 | 0 |
| resiliency_6a | How many friends that are girls do you have? | 28 | 0 |
| resiliency_6b | How many CLOSE friends that are girls do you have? | 33 | 0 |
| accult_phenx_q1_p | How well do you speak English? | 65 | 10 |
| accult_phenx_q2_p | Besides English, do you speak or understand another language or dialect? | 6 | 0 |
| cbcl_q01_p | Acts too young for his/her age. | 6 | 0 |
| cbcl_q03_p | Argues a lot. | 6 | 0 |
| cbcl_q04_p | Fails to finish things he/she starts. | 6 | 0 |
| cbcl_q05_p | There is very little he/she enjoys. | 6 | 0 |
| cbcl_q06_p | Bowel movements outside toilet. | 6 | 0 |
| cbcl_q07_p | Bragging, boasting. | 6 | 0 |
| cbcl_q08_p | Can't concentrate, can't pay attention for long. | 6 | 0 |
| cbcl_q09_p | Can't get his/her mind off certain thoughts; obsessions. | 6 | 0 |
| cbcl_q100_p | Trouble sleeping. | 7 | 0 |
| cbcl_q101_p | Truancy, skips school. | 7 | 0 |
| cbcl_q102_p | Underactive, slow moving, or lacks energy. | 7 | 0 |
| cbcl_q103_p | Unhappy, sad, or depressed. | 7 | 0 |
| cbcl_q104_p | Unusually loud. | 7 | 0 |
| cbcl_q106_p | Vandalism. | 7 | 0 |
| cbcl_q107_p | Wets self during the day. | 7 | 0 |
| cbcl_q108_p | Wets the bed. | 7 | 0 |
| cbcl_q109_p | Whining. | 7 | 0 |
| cbcl_q10_p | Can't sit still, restless, or hyperactive. | 6 | 0 |
| cbcl_q110_p | Wishes to be of opposite sex. | 7 | 0 |
| cbcl_q111_p | Withdrawn, doesn't get involved with others. | 7 | 0 |
| cbcl_q112_p | Worries. | 7 | 0 |
| cbcl_q11_p | Clings to adults or too dependent. | 6 | 0 |
| cbcl_q12_p | Complains of loneliness. | 6 | 0 |
| cbcl_q13_p | Confused or seems to be in a fog. | 7 | 0 |
| cbcl_q14_p | Cries a lot. | 7 | 0 |
| cbcl_q15_p | Cruel to animals. | 7 | 0 |
| cbcl_q16_p | Cruelty, bullying, or meanness to others. | 7 | 0 |
| cbcl_q17_p | Daydreams or gets lost in his/her thoughts. | 7 | 0 |
| cbcl_q19_p | Demands a lot of attention. | 7 | 0 |
| cbcl_q20_p | Destroys his/her own things. | 7 | 0 |
| cbcl_q21_p | Destroys things belonging to his/her family or others. | 8 | 0 |
| cbcl_q22_p | Disobedient at home. | 7 | 0 |
| cbcl_q23_p | Disobedient at school. | 7 | 0 |
| cbcl_q24_p | Doesn't eat well. | 7 | 0 |
| cbcl_q25_p | Doesn't get along with other kids. | 7 | 0 |
| cbcl_q26_p | Doesn't seem to feel guilty after misbehaving. | 7 | 0 |
| cbcl_q27_p | Easily jealous. | 7 | 0 |
| cbcl_q28_p | Breaks rules at home, school or elsewhere. | 7 | 0 |
| cbcl_q29_p | Fears certain animals, situations, or places, other than school. | 7 | 0 |
| cbcl_q30_p | Fears going to school. | 7 | 0 |
| cbcl_q31_p | Fears he/she might think or do something bad. | 7 | 0 |
| cbcl_q32_p | Feels he/she has to be perfect. | 7 | 0 |
| cbcl_q33_p | Feels or complains that no one loves him/her. | 7 | 0 |
| cbcl_q34_p | Feels others are out to get him/her. | 7 | 0 |
| cbcl_q35_p | Feels worthless or inferior. | 7 | 0 |
| cbcl_q36_p | Gets hurt a lot, accident prone. | 7 | 0 |
| cbcl_q37_p | Gets in many fights. | 7 | 0 |
| cbcl_q38_p | Gets teased a lot. | 7 | 0 |
| cbcl_q39_p | Hangs around with others who get in trouble. | 7 | 0 |
| cbcl_q40_p | Hears sound or voices that aren't there. | 7 | 0 |
| cbcl_q41_p | Impulsive or acts without thinking. | 7 | 0 |
| cbcl_q42_p | Would rather be alone than with others. | 7 | 0 |
| cbcl_q43_p | Lying or cheating. | 7 | 0 |
| cbcl_q44_p | Bites fingernails. | 7 | 0 |
| cbcl_q45_p | Nervous, highstrung, or tense. | 7 | 0 |
| cbcl_q46_p | Nervous movements or twitching. | 7 | 0 |
| cbcl_q47_p | Nightmares. | 7 | 0 |
| cbcl_q48_p | Not liked by other kids. | 7 | 0 |
| cbcl_q49_p | Constipated, doesn't move bowels. | 7 | 0 |
| cbcl_q50_p | Too fearful or anxious. | 7 | 0 |
| cbcl_q51_p | Feels dizzy or lightheaded. | 7 | 0 |
| cbcl_q52_p | Feels too guilty. | 7 | 0 |
| cbcl_q53_p | Overeating. | 7 | 0 |
| cbcl_q54_p | Overtired without good reason. | 7 | 0 |
| cbcl_q55_p | Overweight. | 7 | 0 |
| cbcl_q56a_p | Aches or pains (not stomach or headaches). | 7 | 0 |
| cbcl_q56b_p | Headaches. | 7 | 0 |
| cbcl_q56c_p | Nausea, feels sick. | 7 | 0 |
| cbcl_q56d_p | Problems with eyes (not if corrected by glasses). | 7 | 0 |
| cbcl_q56e_p | Rashes or other skin problems. | 7 | 0 |
| cbcl_q56f_p | Stomachaches. | 7 | 0 |
| cbcl_q56g_p | Vomiting, throwing up. | 7 | 0 |
| cbcl_q56h_p | Other (physical problems without known physical cause). | 7 | 0 |
| cbcl_q57_p | Physically attacks people. | 7 | 0 |
| cbcl_q58_p | Picks nose, skin, or other parts of body. | 7 | 0 |
| cbcl_q59_p | Plays with own sex parts in public. | 7 | 0 |
| cbcl_q60_p | Plays with own sex parts too much. | 7 | 0 |
| cbcl_q61_p | Poor school work. | 7 | 0 |
| cbcl_q62_p | Poorly coordinated or clumsy. | 7 | 0 |
| cbcl_q63_p | Prefers being with older kids. | 7 | 0 |
| cbcl_q64_p | Prefers being with younger kids. | 7 | 0 |
| cbcl_q65_p | Refuses to talk. | 7 | 0 |
| cbcl_q66_p | Repeats certain acts over and over; compulsions. | 7 | 0 |
| cbcl_q67_p | Runs away from home. | 7 | 0 |
| cbcl_q68_p | Screams a lot. | 7 | 0 |
| cbcl_q69_p | Secretive, keeps things to self. | 7 | 0 |
| cbcl_q70_p | Sees things that aren't there. | 7 | 0 |
| cbcl_q71_p | Self-conscious or easily embarrassed. | 7 | 0 |
| cbcl_q72_p | Sets fires. | 7 | 0 |
| cbcl_q73_p | Sexual problems. | 7 | 0 |
| cbcl_q74_p | Showing off or clowning. | 7 | 0 |
| cbcl_q75_p | Too shy or timid. | 7 | 0 |
| cbcl_q76_p | Sleeps less than most kids. | 7 | 0 |
| cbcl_q77_p | Sleeps more than most kids during day and/or night. | 7 | 0 |
| cbcl_q78_p | Inattentive or easily distracted. | 7 | 0 |
| cbcl_q79_p | Speech problem. | 7 | 0 |
| cbcl_q80_p | Stares blankly. | 7 | 0 |
| cbcl_q81_p | Steals at home. | 7 | 0 |
| cbcl_q82_p | Steals outside the home. | 7 | 0 |
| cbcl_q83_p | Stores up too many things he/she doesn't need. | 7 | 0 |
| cbcl_q84_p | Strange behavior. | 7 | 0 |
| cbcl_q85_p | Strange ideas. | 7 | 0 |
| cbcl_q86_p | Stubborn, sullen, or irritable. | 7 | 0 |
| cbcl_q87_p | Sudden changes in mood or feelings. | 7 | 0 |
| cbcl_q88_p | Sulks a lot. | 7 | 0 |
| cbcl_q89_p | Suspicious. | 7 | 0 |
| cbcl_q90_p | Swearing or obscene language. | 7 | 0 |
| cbcl_q92_p | Talks or walks in sleep. | 7 | 0 |
| cbcl_q93_p | Talks too much. | 7 | 0 |
| cbcl_q94_p | Teases a lot. | 7 | 0 |
| cbcl_q95_p | Temper tantrums or hot temper. | 7 | 0 |
| cbcl_q96_p | Thinks about sex too much. | 7 | 0 |
| cbcl_q97_p | Threatens people. | 7 | 0 |
| cbcl_q98_p | Thumb-sucking. | 7 | 0 |
| ksads_ptsd_raw_758_p | Witnessed or present during an act of terrorism (e.g., Boston marathon bombing). | 414 | 0 |
| ksads_ptsd_raw_760_p | Witnessed someone shot or stabbed in the community. | 414 | 0 |
| ksads_ptsd_raw_761_p | Shot, stabbed, or beaten brutally by a non-family member. | 414 | 0 |
| ksads_ptsd_raw_762_p | Shot, stabbed, or beaten brutally by a grown up in the home. | 414 | 0 |
| ksads_ptsd_raw_763_p | Beaten to the point of having bruises by a grown up in the home. | 414 | 0 |
| ksads_ptsd_raw_765_p | A family member threatened to kill your child. | 414 | 0 |
| ksads_ptsd_raw_766_p | Witness the grownups in the home push, shove or hit one another. | 414 | 0 |
| ksads_ptsd_raw_767_p | A grown up in the home touched your child in his or her privates, had your child touch their privates, or did other sexual things to your child. | 414 | 0 |
| ksads_ptsd_raw_768_p | An adult outside your family touched your child in his or her privates, had your child touch their privates or did other sexual things to your child. | 414 | 0 |
| ksads_ptsd_raw_769_p | A peer forced your child to do something sexually. | 414 | 0 |
| ksads_ptsd_raw_770_p | Learned about the sudden unexpected death of a loved one. | 414 | 0 |
| medinv_plus_caff_24_p | Has your son/daughter consumed a caffeinated beverage (such as Mountain Dew, Coke, Pepsi, Barq's root beer, energy drink like Red Bull or Monster, coffee or espresso or green tea, black tea, or sweet tea) in the last 24 hours? | 84 | 0 |
| neighb_phenx_1r_p | I feel safe walking in my neighborhood, day or night. | 10 | 0 |
| neighb_phenx_2r_p | Violence is not a problem in my neighborhood. | 23 | 0 |
| neighb_phenx_3r_p | My neighborhood is safe from crime. | 38 | 0 |
| pgbi_1_p | Has your child experienced periods of several days or more when, although he/she was feeling unusually happy and intensely energetic (clearly more than your child's usual self), he/she was also physically restless, unable to sit still, and had to keep moving or jumping from one activity to another? | 6 | 0 |
| pgbi_10_p | Has your child had times when his/her thoughts and ideas came so fast that he/she couldn't get them all out, or they came so quickly others complained that they couldn't keep up with your child's ideas? | 7 | 0 |
| pgbi_2_p | Have there been periods of several days or more when your child's friends or other family members told you that your child seemed unusually happy or high - clearly different from his/her usual self or from a typical good mood? | 6 | 0 |
| pgbi_3_p | Has your child's mood or energy shifted rapidly back and forth from happy to sad or high to low? | 7 | 0 |
| pgbi_4_p | Has your child had periods of extreme happiness and intense energy that last several days or more when he/she also felt more anxious or tense (jittery, nervous, uptight) than usual (other than relates to the menstrual cycle)? | 7 | 0 |
| pgbi_5_p | Have there been times of several days or more when, although your child was feeling unusually happy and intensely energetic (clearly more than his/her usual self), he/she also had to struggle very hard to control inner feelings of rage or an urge to smash or destroy things? | 7 | 0 |
| pgbi_6_p | Has your child had periods of extreme happiness and intense energy (clearly more than his/her normal self) when, for several days or more, it took him/her over an hour to get to sleep at night? | 7 | 0 |
| pgbi_7_p | Have you ever found that your child's feelings or energy are generally up or down, but rarely in the middle? | 7 | 0 |
| pgbi_8_p | Has your child had periods lasting several days or more when he/she felt depressed or irritable, and then other periods of several days or more when he/she felt extremely high, elated, and overflowing with energy? | 7 | 0 |
| pgbi_9_p | Have there been periods when, although your child was feeling unusually happy and intensely energetic, almost everything got on his/her nerves and made him/her irritable or angry (other than related to the menstrual cycle)? | 7 | 0 |
| screentime_1_hours_p | On a typical WEEKDAY, how much TIME does your child spend on a computer, cellphone, tablet, or other electronic device? | 16 | 0 |
| screentime_2_hours_p | On a typical WEEKEND DAY, how much TIME does your child spend on a computer, cellphone, tablet, or other electronic device? | 16 | 0 |
| parental_monitoring_q1 | How often do your parents/guardians know where you are? | 23 | 0 |
| parental_monitoring_q2 | How often do your parents know who you are with when you are not at school and away from home? | 25 | 0 |
| parental_monitoring_q3 | If you are at home when your parents or guardians are not, how often do you know how to get in touch with them? | 24 | 0 |
| parental_monitoring_q4 | How often do you talk to your mom/dad or guardian about your plans for the coming day, such as your plans about what will happen at school or what you are going to do with friends? | 23 | 0 |
| parental_monitoring_q5 | In an average week, how many times do you and your parents/guardians, eat dinner together? | 23 | 0 |
| pea_wiscv_tss | WISC-V Matrix Reasoning Total Scaled Score | 261 | 0 |
| prodrom_psych_10 | Did you lose concentration because you noticed sounds in the distance that you usually don't hear? | 15 | 0 |
| prodrom_psych_11 | Although you could not see anything or anyone, did you suddenly start to feel that an invisible energy, creature, or some person was around you? | 15 | 0 |
| prodrom_psych_12 | Did you start to worry at times that your mind was trying to trick you or was not working right? | 15 | 0 |
| prodrom_psych_13 | Did you feel that the world is not real, you are not real, or that you are dead? | 15 | 0 |
| prodrom_psych_14 | Did you feel confused because something you experienced didn't seem real or it seemed imaginary to you? | 15 | 0 |
| prodrom_psych_15 | Did you honestly believe in things that other people would say are unusual or weird? | 16 | 0 |
| prodrom_psych_16 | Did you feel that parts of your body had suddenly changed or worked differently than before; like your legs had suddenly turned to something else or your nose could suddenly smell things you'd never actually smelled before? | 15 | 0 |
| prodrom_psych_17 | Did you feel that sometimes your thoughts were so strong you could almost hear them, as if another person, NOT you, spoke them? | 15 | 0 |
| prodrom_psych_18 | Did you feel that other people might want something bad to happen to you or that you could not trust other people? | 15 | 0 |
| prodrom_psych_19 | Did you suddenly start to see unusual things that you never saw before like flashes, flames, blinding light, or shapes floating in front of you? | 15 | 0 |
| prodrom_psych_1 | Did places that you know well, such as your bedroom, or other rooms in your home, your classroom or school yard, suddenly seem weird, strange or confusing to you; like not the real world? | 15 | 0 |
| prodrom_psych_20 | Have you seen things that other people can't see or don't seem to see? | 14 | 0 |
| prodrom_psych_21 | Did you suddenly start to notice that people sometimes had a hard time understanding what you were saying, even though they used to understand you well? | 15 | 0 |
| prodrom_psych_2 | Did you hear strange sounds that you never noticed before like banging, clicking, hissing, clapping, or ringing in your ears? | 15 | 0 |
| prodrom_psych_3 | Do things that you see appear different from the way they usually do (brighter or duller, larger or smaller, or changed in some other way)? | 15 | 0 |
| prodrom_psych_4 | Did you feel like you had special, unusual powers like you could make things happen by magic, or that you could magically know what was inside another person's mind, or magically know what was going to happen in the future when other people could not? | 14 | 0 |
| prodrom_psych_5 | Did you feel that someone else, who is not you, has taken control over the private, personal, thoughts or ideas inside your head? | 15 | 0 |
| prodrom_psych_6 | Did you suddenly find it hard to figure out how to say something quickly and easily so that other people would understand what you meant? | 15 | 0 |
| prodrom_psych_7 | Did you ever feel very certain that you have very special abilities or magical talents that other people do not have? | 14 | 0 |
| prodrom_psych_8 | Did you suddenly feel that you could not trust other people because they seemed to be watching you or talking about you in an unfriendly way? | 15 | 0 |
| prodrom_psych_9 | Do you sometimes get strange feelings on or just beneath your skin, like bugs crawling? | 15 | 0 |
| school_risk_phenx_10 | There are lots of chances to be part of class discussions or activities. | 27 | 0 |
| school_risk_phenx_12 | In general, I like school a lot. | 26 | 0 |
| school_risk_phenx_15 | Usually, school bores me. | 26 | 0 |
| school_risk_phenx_17 | Getting good grades is not so important to me. | 27 | 0 |
| school_risk_phenx_2 | In my school, students have lots of chances to help decide things like class activities and rules. | 26 | 0 |
| school_risk_phenx_3 | I get along with my teachers. | 27 | 0 |
| school_risk_phenx_4 | My teacher(s) notices when I am doing a good job and lets me know about it. | 27 | 0 |
| school_risk_phenx_5 | There are lots of chances for students in my school to get involved in sports, clubs, or other school activities outside of class. | 26 | 0 |
| school_risk_phenx_6 | I feel safe at my school. | 27 | 0 |
| school_risk_phenx_7 | The school lets my parents know when I have done something well. | 28 | 0 |
| school_risk_phenx_8 | I like school because I do well in class. | 27 | 0 |
| school_risk_phenx_9 | I feel I'm just as smart as other kids my age. | 27 | 0 |
| macvs_ss_fo_p | MACVS Family Obligation Subscale, Mean. | 7 | 0 |
| macvs_ss_fr_p | MACVS Family as Referent Subscale, Mean. | 7 | 0 |
| macvs_ss_fs_p | MACVS Family Support Subscale, Mean. | 7 | 0 |
| macvs_ss_isr_p | MACVS Independence & Self-Reliance Subscale, Mean. | 7 | 0 |
| meim_ss_com_p | MEIM-R Commitment and Attachment Subscale, Mean. | 691 | 0 |
| meim_ss_exp_p | MEIM-R Exploration Subscale, Mean. | 691 | 0 |
| crpbi_ss_studycaregiver | CRPBI - Acceptance Subscale Mean of Report by Parent Completing Protocol by Youth. | 36 | 0 |
| stq_y_ss_weekday | Screen Time Youth: Weekday Sum. | 25 | 0 |
| stq_y_ss_weekend | Screen Time Youth: Weekend Sum. | 27 | 0 |
| sds_p_ss_da | Disorder of Arousal (DA) Sum. | 6 | 0 |
| sds_p_ss_dims | Disorders of Initiating and Maintaining Sleep (DIMS) Sum. | 6 | 0 |
| sds_p_ss_does | Disorders of Excessive Somnolence (DOES) Sum. | 7 | 0 |
| sds_p_ss_sbd | Sleep Breathing disorders (SBD) Sum. | 6 | 0 |
| sds_p_ss_swtd | Sleep-Wake transition Disorders (SWTD) Sum. | 33 | 0 |
| devhx_ss_12_p | About how many weeks premature was the child when they were born? | 173 | 0 |
| anthro_weight_calc | Average Measured Weight (lbs). | 11 | 0 |
| ehi_ss_score | Handedness score rating | 0 | 0 |
| fes_q1 | We fight a lot in our family. | 26 | 0 |
| fes_q2 | Family members rarely become openly angry. | 26 | 0 |
| fes_q3 | Family members sometimes get so angry they throw things. | 26 | 0 |
| fes_q4 | Family members hardly ever lose their tempers. | 26 | 0 |
| fes_q5 | Family members often criticize each other. | 26 | 0 |
| fes_q6 | Family members sometimes hit each other. | 26 | 0 |
| fes_q7 | If there's a disagreement in our family, we try hard to smooth things over and keep the peace. | 26 | 0 |
| fes_q8 | Family members often try to one-up or outdo each other. | 26 | 0 |
| fes_q9 | In our family, we believe you don't ever get anywhere by raising your voice. | 26 | 0 |
| nihtbx_cardsort_agecorrected | NIH Toolbox Dimensional Change Card Sort Test Ages 8-11 v2.0 Age-Corrected Standard Score. | 162 | 0 |
| nihtbx_cryst_agecorrected | Crystallized Composite Age-Corrected Standard Score. | 353 | 0 |
| nihtbx_flanker_agecorrected | NIH Toolbox Flanker Inhibitory Control and Attention Test Ages 8-11 v2.0 Age-Corrected Standard Score. | 163 | 0 |
| nihtbx_fluidcomp_agecorrected | Cognition Fluid Composite Age-Corrected Standard Score. | 408 | 0 |
| nihtbx_list_agecorrected | NIH Toolbox List Sorting Working Memory Test Age 7+ v2.0 Age-Corrected Standard Score. | 206 | 0 |
| nihtbx_pattern_agecorrected | NIH Toolbox Pattern Comparison Processing Speed Test Age 7+ v2.0 Age-Corrected Standard Score. | 181 | 0 |
| nihtbx_picture_agecorrected | NIH Toolbox Picture Sequence Memory Test Age 8+ Form A v2.0 Age-Corrected Standard Score. | 169 | 0 |
| nihtbx_picvocab_agecorrected | NIH Toolbox Picture Vocabulary Test Age 3+ v2.0 Age-Corrected Standard Score. | 157 | 0 |
| nihtbx_reading_agecorrected | NIH Toolbox Oral Reading Recognition Test Age 3+ v2.0 Age-Corrected Standard Score. | 171 | 0 |
| screentime_wknd_10 | On a typical weekend, how many hours do you: Text on a cell phone, tablet, or computer (GChat, Whatsapp, etc.)? | 29 | 0 |
| screentime_wknd_11 | On a typical weekend, how many hours do you: Visit social networking sites like Facebook, Twitter, Instagram, etc.? | 30 | 0 |
| screentime_wknd_12 | On a typical weekend, how many hours do you: Video chat (Skype, Facetime, etc.)? | 37 | 0 |
| screentime_wkdy_1 | On a typical weekday, how many hours do you: Watch TV shows or movies? | 27 | 0 |
| screentime_wkdy_2 | On a typical weekday, how many hours do you: Watch videos (such as YouTube)? | 30 | 0 |
| screentime_wkdy_3 | On a typical weekday, how many hours do you: Play video games on a computer, console, phone or other device (Xbox, Play Station, iPad)? | 30 | 0 |
| screentime_wkdy_4 | On a typical weekday, how many hours do you: Text on a cell phone, tablet, or computer (e.g. GChat, Whatsapp, etc.)? | 27 | 0 |
| screentime_wkdy_5 | On a typical weekday, how many hours do you: Visit social networking sites like Facebook, Twitter, Instagram, etc.? | 28 | 0 |
| screentime_wknd_7 | On a typical weekend, how many hours do you: Watch TV shows or movies? | 32 | 0 |
| screentime_wknd_8 | On a typical weekend, how many hours do you: Watch videos (such as YouTube)? | 29 | 0 |
| screentime_wknd_9 | On a typical weekend, how many hours do you: Play video games on a computer, console, phone or other device (Xbox, Play Station, iPad)? | 32 | 0 |
| screentime_wkdy_6 | On a typical weekday, how many hours do you: Video chat (Skype, Facetime, etc.)? | 33 | 0 |
| physical_activity_1 | During the past 7 days, on how many days were you physically active for a total of at least 60 minutes per day? (Add up all the time you spent in any kind of physical activity that increased your heart rate and made you breathe hard some of the time) | 31 | 0 |
| physical_activity_2 | On how many of the past 7 days did you do exercises to strengthen or tone your muscles, such as push-ups, sit-ups, or weight lifting? | 36 | 0 |
| physical_activity_5 | In an average week when you are in school, on how many days do you go to physical education (PE) class? | 35 | 0 |
| bis_y_ss_bas_drive | BIS/BAS: BAS Drive. | 26 | 0 |
| bis_y_ss_bas_fs | BIS/BAS: BAS Fun Seeking. | 26 | 0 |
| bis_y_ss_basm_drive | BIS/BAS: BAS Drive (Modified). | 26 | 0 |
| bis_y_ss_bas_rr | BIS/BAS: BAS Reward Responsiveness. | 26 | 0 |
| bis_y_ss_bis_sum | BIS/BAS: BIS Sum. | 25 | 0 |
| upps_y_ss_lack_of_perseverance | UPPS: Lack of Perseverance (GSSF). | 26 | 0 |
| upps_y_ss_lack_of_planning | UPPS-P for Children Short Form (ABCD-version), Lack of Planning. | 26 | 0 |
| upps_y_ss_negative_urgency | UPPS-P for Children Short Form (ABCD-version), Negative Urgency. | 26 | 0 |
| upps_y_ss_positive_urgency | UPPS-P for Children Short Form (ABCD-version), Positive Urgency. | 26 | 0 |
| upps_y_ss_sensation_seeking | UPPS-P for Children Short Form (ABCD-version), Sensation Seeking. | 26 | 0 |
| prosocial_q1 | I try to be nice to other people. I care about their feelings. | 32 | 0 |
| prosocial_q2 | I am helpful if someone is hurt, upset, or feeling sick. | 33 | 0 |
| prosocial_q3 | I often offer to help others (parents, teachers, children). | 33 | 0 |
| married.bl | Parents married? | 98 | 0 |
| kid_adhd_meds | Taking ADHD-related medication(s). | NA | NA |
| kid_depr_meds | Taking depression-related medication(s). | NA | NA |
| activity_phys | Number of physical activities/hobbies (e.g. sports). | NA | NA |
| activity_create | Number of creative activities/hobbies (e.g. music, art). | NA | NA |
| activity_other | Number of other activities/hobbies (e.g. collecting). | NA | NA |
| activity_none | No other activities/hobbies. | NA | NA |
| abcd_site.site01 | ABCD data collection site 1 | NA | NA |
| abcd_site.site02 | ABCD data collection site 2 | NA | NA |
| abcd_site.site03 | ABCD data collection site 3 | NA | NA |
| abcd_site.site04 | ABCD data collection site 4 | NA | NA |
| abcd_site.site05 | ABCD data collection site 5 | NA | NA |
| abcd_site.site06 | ABCD data collection site 6 | NA | NA |
| abcd_site.site07 | ABCD data collection site 7 | NA | NA |
| abcd_site.site08 | ABCD data collection site 8 | NA | NA |
| abcd_site.site09 | ABCD data collection site 9 | NA | NA |
| abcd_site.site10 | ABCD data collection site 10 | NA | NA |
| abcd_site.site11 | ABCD data collection site 11 | NA | NA |
| abcd_site.site12 | ABCD data collection site 12 | NA | NA |
| abcd_site.site13 | ABCD data collection site 13 | NA | NA |
| abcd_site.site14 | ABCD data collection site 14 | NA | NA |
| abcd_site.site15 | ABCD data collection site 15 | NA | NA |
| abcd_site.site16 | ABCD data collection site 16 | NA | NA |
| abcd_site.site17 | ABCD data collection site 17 | NA | NA |
| abcd_site.site18 | ABCD data collection site 18 | NA | NA |
| abcd_site.site19 | ABCD data collection site 19 | NA | NA |
| abcd_site.site20 | ABCD data collection site 20 | NA | NA |
| abcd_site.site21 | ABCD data collection site 21 | NA | NA |
| abcd_site.site22 | ABCD data collection site 22 | NA | NA |
| race.ethnicity.5level.White | What race do you consider yourself to be? White. | NA | NA |
| race.ethnicity.5level.Hispanic | What race do you consider yourself to be? Hispanic. | NA | NA |
| race.ethnicity.5level.Black | What race do you consider yourself to be? Black. | NA | NA |
| race.ethnicity.5level.Asian | What race do you consider yourself to be? Asian. | NA | NA |
| race.ethnicity.5level.Other | What race do you consider yourself to be? Other/mixed. | NA | NA |
| ksads_back_sex_orient.Yes | Are you gay or bisexual? Yes. | NA | NA |
| ksads_back_sex_orient.Maybe | Are you gay or bisexual? Maybe. | NA | NA |
| ksads_back_sex_orient.No | Are you gay or bisexual? No. | NA | NA |
| ksads_back_sex_orient.I.do.not.understand.this.question | Are you gay or bisexual? I do not understand the question. | NA | NA |
| ksads_back_trans_id.Yes | Are you transgender? Yes. | NA | NA |
| ksads_back_trans_id.Maybe | Are you transgender? Maybe. | NA | NA |
| ksads_back_trans_id.No | Are you transgender? No. | NA | NA |
| ksads_back_trans_id.I.do.not.understand.this.question | Are you transgender? I do not understand the question. | NA | NA |
| household.income.bl...50K. | Household income below 50k | NA | NA |
| household.income.bl....50K....100K. | Household income between 50k - 100k | NA | NA |
| household.income.bl....100K. | Household income above 100k | NA | NA |
| high.educ.bl...HS.Diploma | Highest education of parents? No high school. | NA | NA |
| high.educ.bl.HS.Diploma.GED | Highest education of parents? High school or GED. | NA | NA |
| high.educ.bl.Some.College | Highest education of parents? Some college. | NA | NA |
| high.educ.bl.Bachelor | Highest education of parents? Bachelor’s degree. | NA | NA |
| high.educ.bl.Post.Graduate.Degree | Highest education of parents? Graduate degree. | NA | NA |
| devhx_9_alcohol_p | Once you/biomom knew you/she were pregnant, where you/biomom using alcohol? | NA | NA |
| devhx_9_coc_crack_p | Once you/biomom knew you/she were pregnant, where you/biomom using cocaine/crack? | NA | NA |
| devhx_9_her_morph_p | Once you/biomom knew you/she were pregnant, where you/biomom using heroin or morphine? | NA | NA |
| devhx_9_marijuana_p | Once you/biomom knew you/she were pregnant, where you/biomom using marijuana? | NA | NA |
| devhx_9_other_drugs_p | Once you/biomom knew you/she were pregnant, where you/biomom using other drugs? | NA | NA |
| devhx_9_oxycont_p | Once you/biomom knew you/she were pregnant, where you/biomom using oxycontin? | NA | NA |
| devhx_9_tobacco_p | Once you/biomom knew you/she were pregnant, where you/biomom using tobacco? | NA | NA |

**S2 Table.** *Features used to generate internalizing and externalizing disorder labels.*

| **Variable Name (ABCD)** | **Variable Description (ABCD)** | **Label** |
| --- | --- | --- |
| ksads_1_843_p | Diagnosis - Persistent Depressive Disorder (Dysthymia) PRESENT F34.1 | internalizing |
| ksads_1_845_p | Diagnosis - Persistent Depressive Disorder (Dysthymia) PAST F34.1 | internalizing |
| ksads_1_844_p | Diagnosis - Persistent Depressive Disorder (Dysthymia) In Partial Remission F34.1 | internalizing |
| ksads_1_840_p | Diagnosis - Major Depressive Disorder Present | internalizing |
| ksads_1_841_p | Diagnosis - Major Depressive Disorder Current in Partial Remission (F32.4) | internalizing |
| ksads_1_842_p | Diagnosis - Major Depressive Disorder Past (F32.9) | internalizing |
| ksads_1_847_p | Diagnosis - Unspecified Depressive Disorder PAST (F32.9) | internalizing |
| ksads_1_846_p | Diagnosis - Unspecified Depressive Disorder Current (F32.9) | internalizing |
| ksads_8_864_p | Diagnosis - Social Anxiety Disorder (F40.10) PAST | internalizing |
| ksads_8_863_p | Diagnosis - Social Anxiety Disorder (F40.10) PRESENT | internalizing |
| ksads_8_912_p | Diagnosis - Other Specified Anxiety Disorder (Social Anxiety Disorder impairment does not meet minimum duration) PAST F41.8 | internalizing |
| ksads_8_911_p | Diagnosis - Other Specified Anxiety Disorder (Social Anxiety Disorder impairment does not meet minimum duration) F41.8 | internalizing |
| ksads_10_914_p | Diagnosis - Other Specified Anxiety Disorder (Generalized Anxiety Disorder impairment does not meet minimum duration) PAST F41.8 | internalizing |
| ksads_10_913_p | Diagnosis - Other Specified Anxiety Disorder (Generalized Anxiety Disorder impairment does not meet minimum duration) F41.8 | internalizing |
| ksads_10_869_p | Diagnosis - Generalized Anxiety Disorder Present (F41.1) | internalizing |
| ksads_10_870_p | Diagnosis - Generalized Anxiety Disorder Past (F41.1) | internalizing |
| ksads_5_857_p | Diagnosis - Panic Disorder (F41.0) PRESENT | internalizing |
| ksads_5_858_p | Diagnosis - Panic Disorder (F41.0) PAST | internalizing |
| ksads_14_856_p | Diagnosis - Unspecified Attention-Deficit/Hyperactivity Disorder (F90.9) | externalizing |
| ksads_14_855_p | Diagnosis - Attention-Deficit/Hyperactivity Disorder IN PARTIAL REMISSION | externalizing |
| ksads_14_853_p | Diagnosis - Attention-Deficit/Hyperactivity Disorder Present | externalizing |
| ksads_14_854_p | Diagnosis - Attention-Deficit/Hyperactivity Disorder Past | externalizing |
| ksads_15_901_p | Diagnosis - Oppositional Defiant Disorder Present F91.3 | externalizing |
| ksads_15_902_p | Diagnosis - Oppositional Defiant Disorder Past F91.3 | externalizing |
| ksads_16_900_p | Diagnosis - Conduct Disorder past adolescent onset (F91.2) | externalizing |
| ksads_16_897_p | Diagnosis - Conduct Disorder present childhood onset (F91.1) | externalizing |
| ksads_16_899_p | Diagnosis - Conduct Disorder past childhood onset (F91.1) | externalizing |
| ksads_16_898_p | Diagnosis - Conduct Disorder present adolescent onset (F91.2) | externalizing |
| ksads_11_917_p | Diagnosis - Obsessive-Compulsive Disorder Present (F42) | internalizing |
| ksads_11_918_p | Diagnosis - Obsessive-Compulsive Disorder Past (F42) | internalizing |

**SF1.** *Transdiagnostic validation of top predictive features.*

To assess whether the features that were used to classify suicidal ideators (SI) from control participants were different between individuals with internalizing or externalizing disorders, chi square tests or ANOVAs were conducted on categorical and continuous variables, respectively. The tests were run within in each group (control, SI, and suicide attempt [SA]). In the figure, cell color represents the p values and values are Cohen’s d effect sizes. The only significant differences between those with internalizing and externalizing disorders included negative and positive urgency, CBCL items of aggression and swearing, and feeling unhappy, sad, or depressed. However, it is important to note that these effects were found only the controls and with small effect sizes. There were no significant differences between those with internalizing and externalizing disorders in the SI or SA groups for any of the top features.


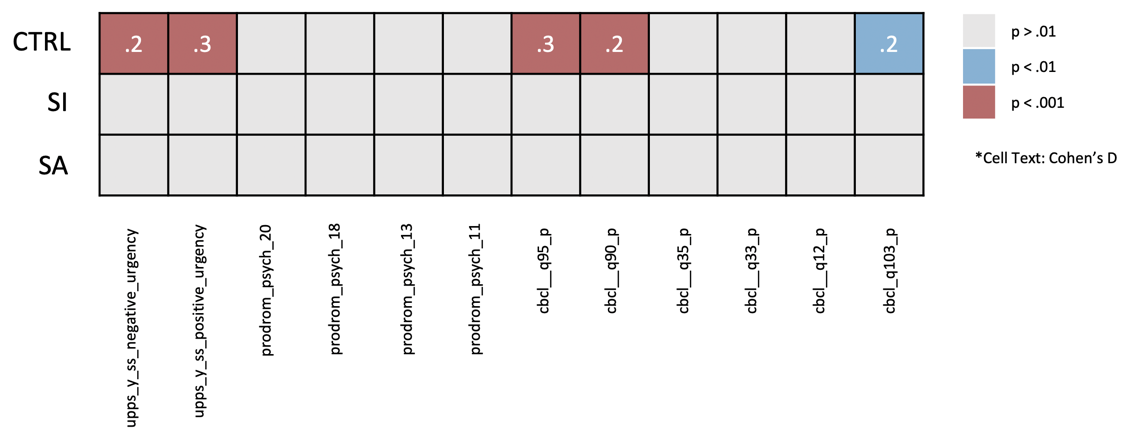


**SF2.** *Performance differences with sibling or random subject removal.*

Model performance in the test set with siblings of participants in the training set removed, or test sets with the same number of individuals removed at random. These two methods examine alterations in performance when removing sibling pairs from train/test sets. This was done to determine if the inclusion of siblings in the original analyses falsely inflated test performance. There were not significant differences between the two removal methods for any of the groups (SI or SA), feature selection methods (none or Boruta), or model types, all p’s > .1.


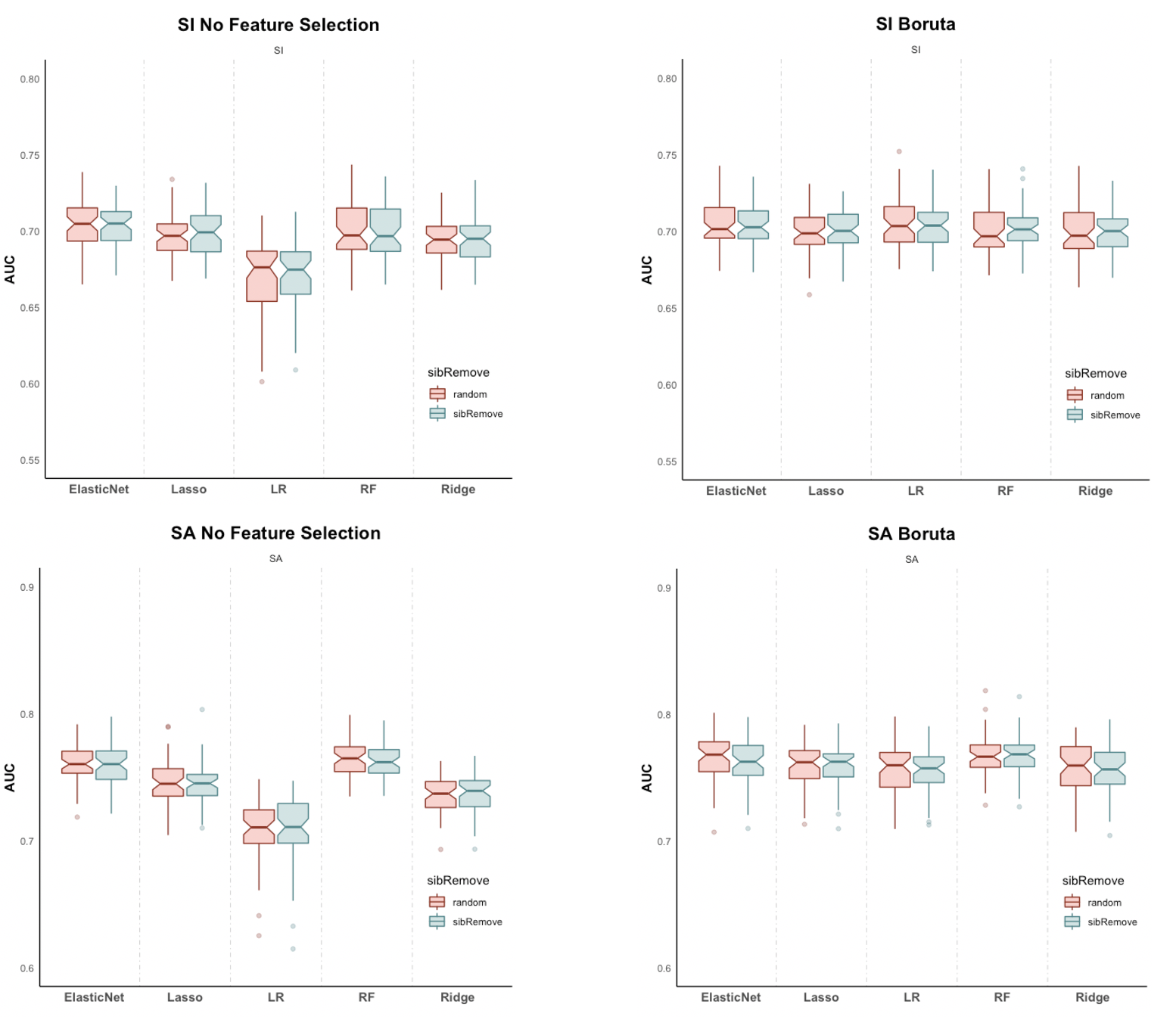

Supplement: S1 File — (DOCX) [file pone.0252114.s001.docx]
